# Rasch analysis and differential item functioning of English language anxiety scale (ELAS) across sex in Egyptian context

**DOI:** 10.1186/s40359-022-00955-w

**Published:** 2022-10-31

**Authors:** Mustafa Ali Khalaf, Ehab Mohammed Naguib Omara

**Affiliations:** 1 Department of Educational Psychology, Minia University, El-Minia, Egypt; 2 Department of Educational Psychology, Ain Shams University, Cairo, Egypt

**Keywords:** English language anxiety, Foreign language classroom anxiety scale, Item response theory, Rasch model, Rasch rating scale

## Abstract

**Background:**

English language anxiety (ELA) is a prevalent phenomenon in language education. It is one of the most commonly investigated non-linguistic variables in studies of language learning. Accordingly, numerous studies have paid great attention to the factors leading to FLA.

**Methods:**

As instruments of ELA have not been subjected to a rigorous test of item response theory (IRT), this study conducted such analysis using the Rasch rating scale model. ELAS scale developed using classical testing theory (CTT), which consists of 32 items measuring four sub-scales (listening, speaking, reading and writing anxiety), was analysed using IRT. WINSTEPS software and SPSS version 26 were used to examine the psychometric properties, sex differential item functioning (DIF) and Rasch analysis of the ELAS in the Egyptian context. A total of 604 participants were recruited for data collection.

**Results:**

The main findings indicated that the scale yielded a good approximation of Rasch assumptions and appears to be a valid and reliable tool. DIF was computed using the Mantel–Haenszel (MH) method and Welch’s t-test, which indicated that no sex bias was detected for any item of the ELAS.

**Conclusions:**

This paper presents a promising ELA instrument characterized by adequate validity, reliability and objectivity in addition to potential for precise use in comparison between males and females because it is invariant across sex.

## Background

The literature on foreign language education considers foreign language anxiety (FLA) a predominant affective factor in language learning (e.g. [1–7]). It is one of the most commonly investigated non-linguistic variables in studies of language learning. Accordingly, numerous studies have paid great attention to the factors leading to FLA [8, 9]. Although many learners and teachers all over the world experience FLA [10], the literature investigating FLA has reported little empirical evidence to declare whether students and teachers of English as a foreign language (EFL) are aware of it [11].

FLA is considered a major problem as it causes different embarrassing situations for many students. The development and validation of reliable and valid measurement tools is a major area of interest within the field of language learning, especially in countries where English is not the official medium of instruction. English language anxiety (ELA) is a classic problem that has been studied by many researchers using measures developed in light of classical testing theory (CTT), although researchers in recent years have shown an increased interest in using item response theory (IRT) to assess the psychometric properties of measurement instruments that were previously developed using CTT.

One of the most widely used measures in this filed is the Horwitz’s Foreign Language Classroom Anxiety Scale (FLCAS), which has been shown to have good psychometric properties according to CTT [3, 12, 13]. The FLCAS has been translated and adapted in different Eastern and Western cultures, including Iran [14], Ethiopia [15], Malaysia [16], Sudan [17], Saudi Arabia [18, 19], Thailand [20], Egypt [4, 21], and China & Pakistan [22]. However, few studies have analysed the FLCAS using the Rasch model [23].

Despite its popularity and wide use in the literature, several authors have criticized the FLCAS and pointed out flaws concerning its validity and reliability. Panayides and Walker [23] have claimed that previous studies yielded a different factor structure for the FLCAS. For example, Aida [1] found a four-factor structure, while other studies extracted two factors [12, 24]. A few years later, Tóth [13] and Bora and Jongmin [25] found that the FLCAS has a unidimensional factor structure. Panayides and Walker [23] concluded that the FLCAS has included many parallel items. They therefore removed five items which poorly fitted Rasch model assumptions and extracted a unidimensional factor structure. These conflicting results related to the factorial structure of the FLCAS raised certain concerns for researchers who use its total score.

As a result, different attempts have therefore been made to develop FLA scales using CTT [26, 27] or scales that assess four brief foreign language skill–specific anxieties [4, 5, 28], such as anxiety scales for reading [29, 30], anxiety scales for speaking [31], anxiety scales for writing [26], and anxiety scales for listening [32, 33]. Other studies have pinpointed “the vital need to develop robust and standardized measurement instrument using IRT for researchers who are interested in the quantitative assessment of foreign language anxiety” [4, 5]. It is obvious that a large body of research has constructed instruments that measure FLA [20, 29, 30, 34, 35]. Nevertheless, researchers have hitherto paid scant attention to the use of Rasch model analysis to develop FLA scales. Little work has been done in the Egyptian context, although English is a mandatory subject for all elementary schooling years [36]. Notwithstanding, there is wide consensus that FLA is a situation-specific of anxiety and represents a complex phenomenon and predicator of foreign language achievement [37].

This creates a demand for the validation of a measure of ELA using the assumptions of IRT—specifically a Rasch model—because measurement tools developed in light of IRT are more accurate [38, 39]. A rigorous psychometric analysis of a new Arabic ELAS in light of IRT is obviously needed in the Egyptian context, where English is increasingly taught and recognized as an official medium of instruction in different private, national and international schools. Accordingly, the purpose of the present study is to provide a Rasch rating scale model analysis of the ELAS and assess its differential item functioning (DIF) across sex in the Egyptian context.

## Rationale for the study

Psychological and educational assessments primarily depend upon valid and reliable measures [40]. Khalilzadeh and Khodi [41] argued that researchers find difficulties in selecting the appropriate scale. They found that scales with the same name might measure concepts that are not the same, and vice versa, scales with different names often measure quite similar concepts [41].

It is well-documented that the use of IRT in validating psychological and educational instruments has led to positive changes in the development of psychological tests [42]. Accordingly, the time has come for the field to embrace measurement instruments developed in the light of IRT, which is in line with the works of Zanon et al. [42] who underestimate the findings and conclusions reached via tools developed using CTT assumptions. Although there have been numerous repeated calls for the use of Rasch-based instruments to assess FLA [4, 5, 7, 43], there has been an unjustified absence of studies that construct tools in accordance with the assumptions of the Rasch model. That is, extensive research has developed instruments for the measurement of ELA in the light of the CTT, while no studies to date have attempted to develop ELA tools using the Rasch rating scale in the Egyptian context.

## ELA in the Egyptian context

Lack of oral practice in teaching and learning English language contributes to the foreign language anxiety among Egyptian undergraduates. Lecturers might place more emphasis on reading and writing activities, and ELA is negatively associated with language performance and achievement [1, 2, 4, 5, 32]. Additionally, the status quo of English education in Egypt neglects oral tasks in language assessment, where the dominant format of assessment is the written exams. Relevant literature in settings other than Egypt supports this claim [2, 20].

It is well-documented in the literature that FLA is a multi-dimensional construct [2, 3, 10]. However, other studies have found that FLA may consist of three dimensions: speaking anxiety, failure anxiety and lack of self-confidence [44]. Lack of self-confidence and fear of making mistakes are possible reasons for speaking anxiety, which tends to appear in shy students who feel uncomfortable because of the communication requirements. Students in most Egyptian EFL classrooms are used to being passive recipients of knowledge. In this vein, Tsiplakides and Keramids [45] posited that “teachers attribute students’ avoidance of speaking situations to reasons such as weak motivation or poor attitude”. Consequently, students rarely speak English in front of their classmates for fear of the teacher’s criticism and peers’ comments. In Egyptian classrooms, students are at a loss for words and worry that they will make mistakes when asked to speak or answer questions using English. Thus, reducing student anxiety and providing a less stressful classroom environment might enable teachers to help students improve both language proficiency as well as overall course performance [32].

According to a seminal study, “language anxiety stems from the inherent inauthenticity associated with immature second language communicative abilities” [3]. Teachers of English in Egypt are mostly unaware of communicative language teaching principles and implementation, because they use traditional approaches that emphasize accuracy rather than fluency; indeed, very few teachers of English in Egypt are proficient in speaking English [46]. For miscellaneous reasons, most language instructors in the Egyptian context do not prepare students for real-world interaction with native speakers and everyday life activities—that is, students are not trained in initiating and engaging in a variety of authentic and communicative language activities. Teachers’ use of traditional approaches and teaching methods, as well as their old-fashioned assessment techniques, are another possible reason for ELA. Crowded classrooms are another potential reason for neglecting speaking and listening activities; lack of facilities and the equipment needed to conduct listening and speaking tasks could also be a strong factor. As a result, listening and speaking skills are shelved by both teachers and students. Lack of student enthusiasm for learning English could create ELA, and most students seem to learn English just to pass exams [4].

## ELA and language performance

Almost 4 decades ago, the leading scholar on FLA, Elaine Horwitz, theorized that the primary components of FLA are communication apprehension, test anxiety and fear of negative evaluation [3]. Recent studies have concluded that the FLA construct is a four skill–based anxiety [4, 5, 26–29, 33].

The role of FLA in the academic achievement and performance of university students has received increasing attention in recent years. A reverse correlation was found between FLA and oral performance—that is, college students with higher FLA scored lower on oral language performance. Additionally, they showed different academically debilitating behaviours such as procrastination, fear of evaluation and excessive concern about errors [35, 47]. A consistent moderate negative correlation was detected between FLA and performance; 25% of the variance in achievement score is explained by FLA and vice versa [1, 2, 10]. More importantly, students who were more highly anxious were more likely to obtain a grade B or lower, while those who were more relaxed were more likely to get a grade (A) ([1]. Undergraduates tend to become more anxious when they have to speak with native speakers [48]. It is worth noting that one-third of American university students experienced moderate to severe levels of FLA, while ELA was negatively and significantly correlated to English Language achievement among seventh graders and college students [49–52].

## Sex differences in ELA

The comparison of factors contributing to FLA between male and female students indicates that the effect of the sex on FLA is still not clearly established in literature. The research results have been contradictory. Although male students report a higher degree of reading anxiety than female students [30], they give less importance to foreign languages than females [49, 53]. In other words, male students were more confident than female students in their FL learning [54]. Female students gained higher scores on the ELA scale than male students [49], and they were more anxiety-ridden than male participants, as they avoided social interaction, probably because they were brought up in a conventional male-dominant society [55]. Female students thus became more anxious than male students during English-speaking exercises in the classroom [56]. Mixed-sex classrooms were regarded as an anxiety-provoking setting, because the presence of the opposite sex in EFL classrooms was found to cause a significant amount of ELA among learners [57]. Other studies found no significant difference between males and females in overall ELA [4, 5, 12, 32, 58, 59]. The findings to date have been conflicting, because the ELA construct is complex and influenced by instructional, societal, cultural and personal factors. A possible reason for the conflicting findings concerning sex differences in ELA can be attributed to the defects in the validity and reliability procedures of the foreign language measurement tools [55]. Few studies have investigated the sex differences in ELA among Egyptian university students [4, 5].

## Rasch rating scale model

Measurement tools are often developed using the assumptions of CTT. In the IRT, “the basic problem in measurement revolves around the connection between the observed data and a measurement model that can be utilized to obtain parameter estimates that reflect person location on a latent variable” [60]. The Rasch model is characterized by simplicity and effectiveness in the construction of measurement tools [39], and Rasch rating scales can replace the CTT in scale construction [42], because IRT-based models provide sound psychometric properties for measurement tools. Another important point to stress is that, unlike the CTT, Rasch analysis converts the ordinal data to equal-interval data, and this facilitates comparison and analysis using the sum score; moreover, persons and items are located on the same continuum using the logit as a common unit of measurement [39]. Because it represents a powerful statistical methodology, IRT is extensively used in psychometric analysis and the calibration of educational and psychological measurement tools [38]. The Rasch model is primarily based on mathematical logits. Through the use of logits instead of raw scores, it is easy to identify the person location on the ability continuum and thus any difference in logits means an equal difference in the latent trait [39].

IRT is based upon robust fundamental assumptions, including unidimensionality, local independence and item characteristic curve. The central idea behind the IRT is “to test whether a higher trait level is associated with a higher probability that a person will endorse this item”. The family of IRT has models for dichotomous item responses and later models for polytomous responses have also been presented. The most widely used models for polytomous responses are the partial credit and Rasch rating scale models [61]. Recently, Rasch analysis has gained increasing importance in language assessment [43].

The Rasch model is a simple and effective tool in the development and validation of self-report rating scales, as it produces valid and reliable instruments with stable measurement properties for both the social sciences and medical research. Rasch analysis enables researchers to rescore, modify, remove items or develop new items and delete specific persons. Person parameters are independent of item parameters. Due to the interval scale scores for persons and measurement invariance across groups produced by Rasch analysis, standardized comparisons are easily administered [39]. As a result, the model has been used to calibrate measures previously constructed using other theoretical frameworks [62]. Those measures can then be used for sound comparisons among persons, because all scientific statements deal with comparisons, and comparisons should be objective [60].

The separation of person and item parameters is one of the most interesting properties of the Rasch model and implies the possibility of estimating a person’s abilities independently from item difficulties and vice versa [63]. One of the strengths of the Rasch model is “its ability to identify differences in the direction of the items even when they measure the same construct” [23]. The measure analysed in the present study uses a 5-point Likert type scale, that is why Rasch rating scale model was used as it is the most appropriate for Likert-type scales [64].

## DIF across sex

DIF refers to the idea that sex, ethnic or age groups respond in different ways, although they possess the same latent trait level [39]. There are two kinds of DIF: uniform and non-uniform. Uniform DIF occurs when the statistical relationship between the item responses and the group of test takers is constant for all levels of the latent trait [65], while non-uniform DIF occurs when “an item discriminates across the levels of ability differently for the groups”; non-uniform DIF “occurs at a lower rate than uniform DIF in practice” [66]. In this context, it is the psychometricians’ responsibility to ensure that the test is fair for all examinees, so it will be “valid for use with students from diverse groups” [66]. According to Milfont and Fischer [67], “the establishment of measurement invariance is a prerequisite for meaningful comparisons across groups”.

Test bias is one of the most problematic issues in measurement tools. Bias towards a certain group of examinees can undermine fairness [68], which is considered essential evidence for the validity of test score interpretations [70]. The calculation of DIF guarantees test fairness and reduces test bias [69]. Bias against sex might be a possible reason for misleading interpretation of sex differences in a given construct or could indicate false differences [7]. DIF is thus a potential source for determining poor test fairness, because its items operate differently across groups, which in turn threatens validity. Accordingly, detection of DIF is an important procedure in the test construction and validation process [71], and the items marked by DIF should be removed [68]. There are many statistical techniques for assessing assess DIF [65]; one of the most popular techniques is the method of Mantel–Haenszel (MH) that has been extensively used in the educational and psychological literature to investigated sex DIF [68].

## Methods

### Method

The main purpose of the article is to reach a psychometrically sound multidimensional scale of ELA in a non-Western setting. The original scale consisted of 48 items developed using CTT assumptions [4]. Confirmatory factor analysis (CFA) resulted in shortening the length of the scale to 32 items. In the present investigation, the Rasch rating scale was adopted to ensure the psychometric properties of the scale using the assumptions of the IRT.

#### Participants

The total sample size was 604 students, with no missing data. In total, 52% of the participants were female and 48% were male. The mean age was 20.43 years (SD = 0.97). Participants were enrolled at Minia University, a public university in North Upper Egypt. The participants have studied English for more than 10 years. All students were informed of the aims and procedures of the study, and their informed consent was documented; their right to withdraw at any time was guaranteed. The simple random sampling technique was adopted to select participants; this is regarded as the most rigorous form of probability sampling from a population. The popular procedure in simple random sampling is to assign a number to each person in the target population and then use a random number table [72]. A complete list of the population was provided by the registration office in the College of Education, and we then selected at random the required number of subjects for the sample from a list of the population. The scale was administered to 650 students out of which 46 forms were excluded due to missing data. The remaining 604 responses were subjected to Rasch analysis. Each selected student participated in the data collection process because the first author was teaching them a course and it was possible to contact them face to face in a weekly lecture. According to Linacre [64] and Jiang et al. [73], sample consisting of 604 will be large enough to calibrate the eight items in each subscale.

#### Measure

The ELAS was developed and validated using CTT assumptions by Khalaf [4]. The scale consisted of 46 items measuring four factors of ELA. This scale was found to be a reliable and valid tool. Exploratory factor analysis (EFA) yielded four factors: speaking anxiety, 15 items, α = 0.93; listening anxiety, 14 items, α = 0.85; writing anxiety, 9 items, α = 0.88; and reading anxiety, 8 items, α = 0.85. Khalaf [5] shortened the scale and reached a brief measure consisting of 32 items. CFA was conducted to ensure structural validity. The results of the CFA indicated that the four-factor model fit well, and the findings were consistent with the criteria for goodness of fit index (GFI) > 0.90 and (RMR < 0.05). Items were measured on a five-point Likert scale ranging from strongly disagree (1) to strongly agree (5). All items were positively worded with no reverse scoring. Sample items for each skill include: *I fear communicating in English* (speaking anxiety); *I find difficulty in written expression* (writing anxiety); *Learning to read in English is a difficult task* (reading anxiety); and *I feel disappointed while answering listening tasks* (listening anxiety)*.* A high score represents greater exposure to ELA-provoking experiences.

### Data analysis

The current study aimed to develop the ELAS using the Rasch rating scale model. The WINSTEPS software was applied for the data analysis using the Rasch model, which allows calibrating polytomous items. To estimate item and person parameters using the Rasch model, a number of pre-requisites should be verified, including unidimensionality of the scale and local independence of their items. Principal component analysis PCA on the standardized residuals SR was used to investigate unidimensionality. CFA was also conducted to investigate the fitting of the one-factor and four-factor structures. The standardized residual correlations were used to check the local independence of items from the ELAS. The infit and outfit mean-squares (which should be between 0.6 and 1.4) were used to assess an item’s fit to the Rasch model. Reliability coefficients and separation indices were estimated for items and persons. Separation indices were used to check that the items discriminate different levels of person performance (“test” reliability) and that persons are able to discriminate differences in item calibration. Values of separation indices > 2 and reliability coefficients > 0.70 are considered adequate [74]. DIF analysis was conducted across sex using the Rasch–Welch test statistics and MH chi square generated by WINSTEPS. The DIF Welch value and MH should be significant to reject the null hypothesis. The Statistical Package for the Social Sciences (SPSS, version 26) was used to compute the descriptive statistics.

## Results

### Descriptive statistics

Table 1 presents the descriptive statistics, which indicate that the scores follow a normal distribution. It is crystal clear that speaking and writing anxieties are higher than listening and reading anxieties. Skewness values range between − 0.36 and 0.16, while the kurtosis values ranged between − 0.72 and − 0.08. Taken together, those data indicate the normal distribution of the participants’ scores. Positive correlations were found among the four subscales of the ELAS. Alpha coefficients range between 0.83 and 0.86, indicating acceptable reliability.

**Table 1:** Mean, standard deviation, skewness, kurtosis and correlations among the four subscales

Factor	M	SD	Skewness	Kurtosis	1	2	3	4
1—Speaking	3.20	0.78	− 0.15	− 0.72	**0.86**	.51**	.39**	.49**
2—Writing	3.54	0.64	− 0.36	− 0.08		**0.83**	.35**	.42**
3—Reading	2.84	0.69	− 0.05	− 0.31			**0.85**	.42**
4—Listening	2.56	0.75	0.16	− 0.60				**0.85**

**Correlation is significant at the p < 0.01 level. The diagonal bold values are the Cronbach’s alpha coefficient

### Rasch analyses: unidimensionality and local independence

**Fig. 1:**
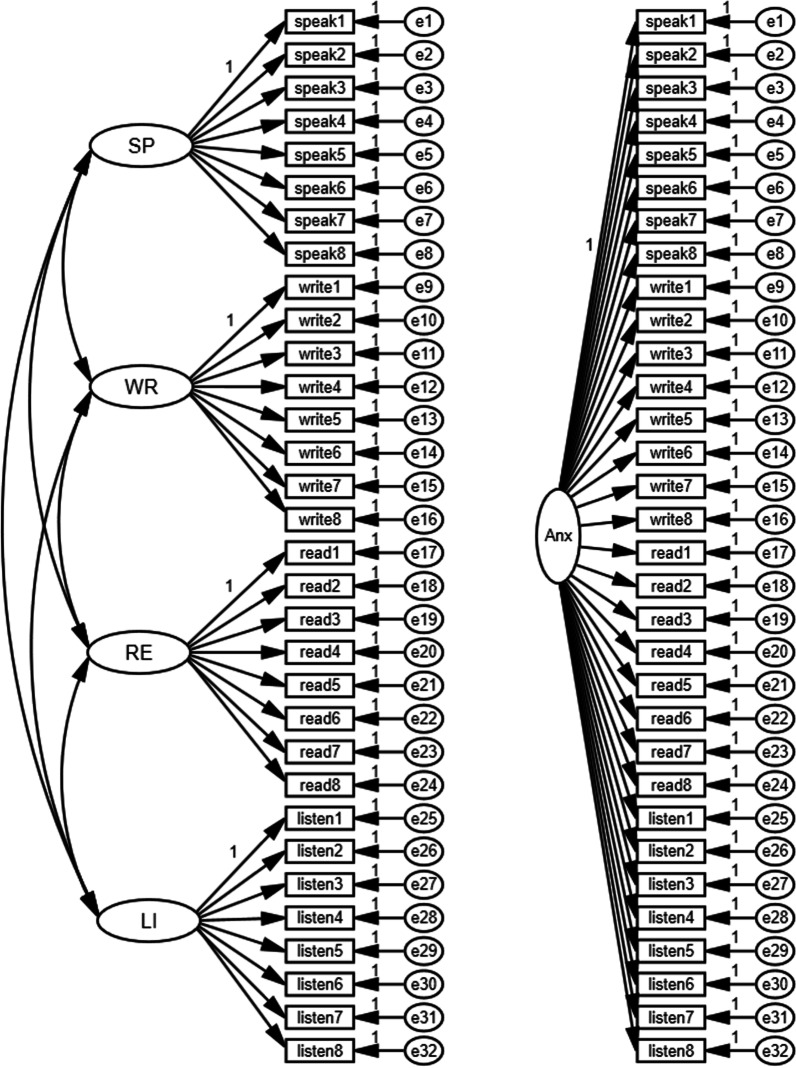
One- and four-factor models for the ELAS

To ensure unidimensionality, as a vital assumption for the use of Rasch model analysis in estimating person and item parameters, two techniques were used: first, PCAR via WINSTEPS software to investigate the dimensionality of the scale. Second, CFA was performed to compare between both the one- and four-factor structural models (see Fig. 1). To check local independence assumption, the second requirement of Rasch model, standardized residual correlations were explored. Local independence is a fundamental assumption of Rasch models; WINSTEPS analysis provides residual item correlations for each item pair.

#### Principal component analysis of the standardized residuals (PCASR)

WINSTEPS was used to perform PCASR for the entire scale (32 items) and then for each separate subscale. The Results reported in Table 2 indicate the total variance explained was 40.7%, while the eigenvalues of the first and second factors were higher than 3, which in turn supports the dimensionality of the scale. The reanalysis of each sub-scale revealed that the total variance explained ranged between 55.2% and 65.75, and the eigenvalue of the first factor was lower than 2, which indicates the absence of any other factor if Rasch analysis was reused with the independent subscales. The first round of the Rasch model analysis revealed that the ELAS is not unidimensional, because the unexplained variance in the first contrast (3.6), second contrast (3.1) and third contrast (2.8) were greater than 2.0 in the PCA.

**Table 2:** Results of principal component analysis of the standardized residuals, separation and reliability values of persons and items

Subscales	Raw variance explained by measure (%)	Unexplained variance			Separation index		Reliability	
		Total	1st contrast	2st contrast	Persons	Items	Persons	Items
Speaking anxiety	15.3 (65.7%)	8.0 (34.3%)	1.6 (6.7%)	1.4 (6.0%)	2.20	12.23	0.83	0.99
Writing anxiety	9.80 (55.2%)	8.0 (44.8%)	1.6 (8.7%)	1.3 (7.5%)	1.98	8.25	0.80	0.99
Reading anxiety	11.1 (58.2%)	8.0 (41.8%)	1.9 (10.0%)	1.4 (7.3%)	2.19	8.07	0.83	0.98
Listening anxiety	13.6 (63.0%)	8.0 (37.0%)	1.6 (7.4%)	1.3 (6.0%)	2.07	8.95	0.81	0.99
Total score	22.0 (40.7%)	32.0 (59.3%)	3.6 (6.7%)	3.1 (5.8%)	3.01	12.79	0.90	0.99

The unexplained variance in the Table 2 is a part of the principal component analysis of the standardized residuals (PCASR). In the current research it is used only to investigate the unidimensionality of the scale.

#### Confirmatory factor analysis

AMOS software version 22 was used to perform CFA to compare between the hypothesized factorial structures of both models given in Fig. 1. Model 1 assumes that the items measure one latent trait (ELA), while model 2 assumes that the scale has a four-factor structure relevant to the four language skills. The findings in Table 3 obviously show that the four-factor model fits the data well, and the goodness of fit indices fall within the acceptable limits (CFI and TLI values were close to 1) while (RMSEA was less than 0.08). The one-factor model statistics fit less well.

**Table 3:** Model fit indices for one- and four-factor models

Model (32 items)	χ^2^	*df*	*p*	CFI	TLI	RMSEA	90% CI for RMSEA	SRMR	BIC
One-factor model	3633.25	464	0.000	0.582	0.554	0.106	0.103–0.110	0.096	4043.08
Four-factor model	1420.88	456	0.000	0.873	0.862	0.059	0.056–0.063	0.051	1881.94

#### Local independence

Local independence represents the mathematical definition of the latent trait [81]. Likewise, Hattie [75] has argued that local independence is a more fundamental concept than unidimensionality, as it implies that the only relation between items is explained by the conditional relationship with the latent variable [75, 76]. It is therefore essential to satisfy this basic assumption of Rasch model analysis. Violation of local independence threatens the psychometric properties of the measurement tools and can lead to misleading data [77]. To investigate the local independence of items, the largest standardized residual correlations (known as Q3 in the psychometrics literature) were explored using WINSTEPS. Certain pairs of items measuring the same factor were found to be correlated (r =  > 0.3), because they assess the same construct, but after we re-analysed the data for each subscale independently, local independence was retained for the four subscales, as the largest standardized residual correlations did not exceed 0.20, the limit indicated in relevant studies [78]. Generally, the previous findings suggest that the scale is multi-dimensional. Thus, adopting this perspective, the authors anticipate that future users of the scale will be able to use separate scores for the four language anxiety subscales instead of using the total score.

### Item fit to the Rasch model

The findings reported in Table 4 show the infit and outfit of the items; the infit values ranged between (0.75:1.58; 0.86:0.32; 0.74:1.35; 0.65:1.34) and outfit vales ranged between (0.76:1.56; 0.85:1.29; 0.74:1.34; 0.64:1.36) for speaking, writing, reading and listening anxieties, respectively. These values fall within the acceptable limits of Rasch analysis (0.60:1.4) [79]. Point bi-serial correlation values among items in each subscale and in the entire scale exceeded 0.5, which indicates the discriminant ability of these items.

**Table 4:** Item calibration, standard error of item calibrations, point-biserial correlations and infit/outfit mean squares generated by WINSTEPS

Items	Item difficulty (measure)	SE	Infit		Outfit		PTME
			MSQ	ZSTD	MSQ	ZSTD	
SP8	0.84	0.05	0.95	− 0.92	0.96	− 0.68	0.74
SP5	0.53	0.05	0.95	− 0.86	0.97	− 0.48	0.70
SP2	0.52	0.05	1.01	0.19	0.99	− 0.09	0.70
SP4	0.47	0.05	1.58	8.98	1.56	8.64	0.64
SP3	0.07	0.05	0.75	− 4.90	0.76	− 4.72	0.72
SP6	− 0.50	0.05	0.98	− 0.37	0.96	− 0.68	0.71
SP7	− 0.93	0.06	0.89	− 1.98	0.87	− 2.39	0.70
SP1	− 1.01	0.06	0.83	− 3.03	0.80	− 3.58	0.73
WR4	0.93	0.06	0.97	− 0.60	0.97	− 0.50	0.71
WR3	0.42	0.06	1.20	3.30	1.21	3.52	0.61
WR8	0.26	0.06	0.86	− 2.59	0.85	− 2.74	0.66
WR6	0.05	0.06	0.94	− 1.12	0.96	− 0.67	0.66
WR7	− 0.07	0.06	1.32	5.15	1.29	4.78	0.59
WR2	− 0.47	0.06	0.86	− 2.56	0.86	− 2.55	0.70
WR5	− 0.54	0.06	0.91	− 1.72	0.88	− 2.16	0.72
WR1	− 0.59	0.06	0.91	− 1.56	0.92	− 1.45	0.70
RE8	0.72	0.06	0.93	− 1.24	0.94	− 1.14	0.68
RE6	0.40	0.06	1.35	5.63	1.34	5.39	0.65
RE3	0.33	0.06	1.07	1.16	1.05	0.95	0.71
RE4	0.26	0.06	0.81	− 3.57	0.80	− 3.71	0.74
RE2	0.03	0.06	0.79	− 4.06	0.78	− 4.22	0.76
RE1	− 0.46	0.06	0.74	− 5.07	0.74	− 5.14	0.74
RE5	− 0.60	0.06	1.03	0.61	1.03	0.56	0.70
RE7	− 0.69	0.06	1.25	4.09	1.25	4.13	0.62
LI3	0.50	0.05	0.82	− 3.31	0.80	− 3.49	0.76
LI4	0.45	0.05	1.09	1.51	1.06	1.03	0.67
LI5	0.34	0.05	0.65	− 7.20	0.64	− 6.92	0.78
LI6	0.18	0.05	1.34	5.55	1.36	5.58	0.57
LI7	0.08	0.05	0.84	− 2.94	0.85	− 2.65	0.77
LI1	0.04	0.05	0.90	− 1.79	0.90	− 1.82	0.68
LI8	− 0.65	0.05	1.12	2.16	1.11	1.91	0.68
LI2	− 0.94	0.05	1.18	3.15	1.19	3.18	0.60

#### Person and item reliability using the Rasch model

Reliability was ensured through the person separation index, which reached 1.98 for writing anxiety (boundary value) and 2.20 for speaking anxiety. Conversely, the item separation index was 8.7 for reading anxiety and 12.23 for speaking anxiety. These findings indicate the discriminant ability for items and persons and the high stability of parameter estimation. The person separation index reached 0.80 for writing anxiety, 0.83 for speaking and reading anxiety, while the reliability of the items in the four subscales exceeded 0.98. Taken together, the values for reliability highlight the ability of the subscales to adequately discriminate among the different levels of the latent trait [74].

#### Person and item calibration

**Fig. 2:**
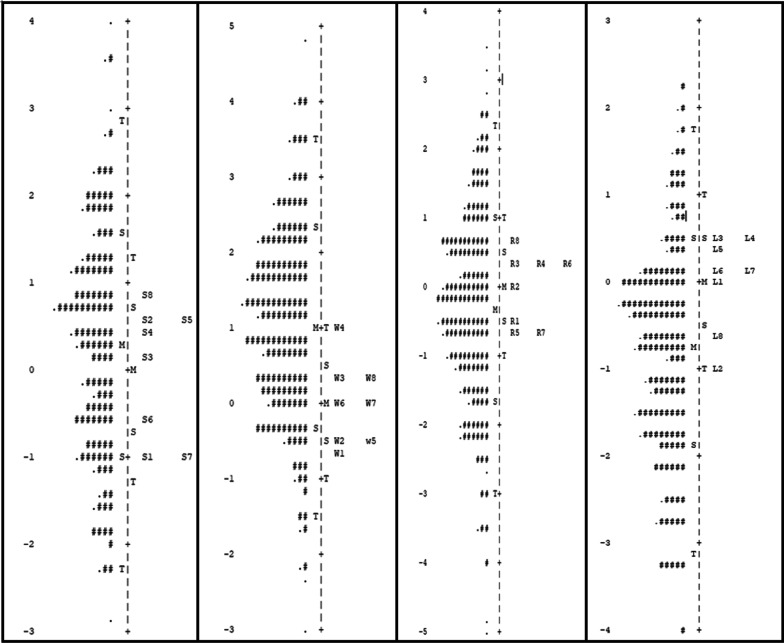
Person-item map of the four subscales after calibration using the Rasch measurement model

For the speaking anxiety subscale, difficulty indices (the amount of the latent trait measured by each item) presented in Table 4 ranged between − 1.01 for item 1 (*I worry when I have to speak in English*) and 0.84 for item 8 (*I fear communicating in English*). For the writing anxiety subscale, difficulty values ranged between − 0.59 for item 1 (*I worry when I write an essay in English*) and 0.93 for item 4 (*I feel mindless when I begin to write in English*). In the reading anxiety subscale, difficulty indices ranged from − 0.69 for item 7 (*Learning to read in English is a difficult task*) to 0.72 for item 8 (*I feel upset when asked to read unfamiliar topics*). Finally, in the listening anxiety subscale, difficulty indices ranged from − 0.94 for item 2 (*I find difficulty in understanding native speakers of English*) to 0.50 for item 3 (*I have difficulty in understanding lectures, news and dialogues delivered in English*). It is clear that the difficulty indices cover the middle area of the latent trait, and the items do not extend the extremes of the construct. The claim is supported by the person-item map of the four subscales, which indicates that item blocks exist at the middle point of the trait continuum. As a result, the ELAS can provide more precise and reliable information for persons with medium levels of ELA than for those with higher or lower levels (Fig. 2).

#### DIF across sex

**Fig. 3:**
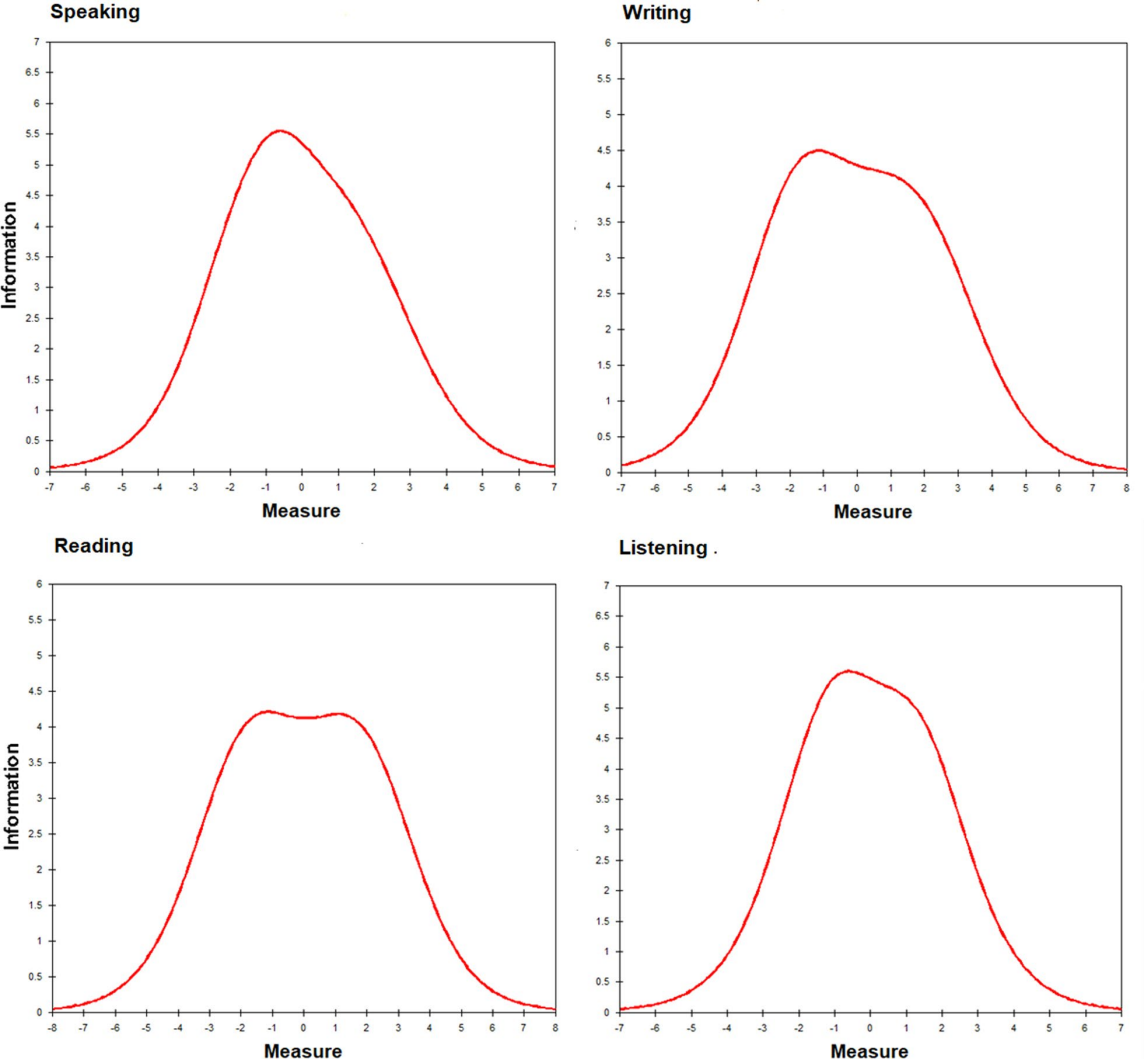
Test information curves of the four subscales

DIF was detected using WINSTEPS Rasch–Welch’s t and MH values. Welch’s t values (see Table 5) were insignificant for all items. Similarly, MH values were insignificant (0.02–2.97; 0–1.93; 0–3.81; and 0.07–2.40) for speaking, writing, reading and listening anxieties, respectively. Taken together, those findings indicate that items do not appear to function differently across sexes, and they assess the latent trait equivalently across both sexes. Figure 3 shows test information curves for the four sub-scales of ELAS.

**Table 5:** Differential item functioning analysis of the items of the four subscales using Rasch–Welch’s t and Mantel–Haenszel chi square indices

Item	Females			Males			DIF CONTRAST	Joint S.E	Rasch–Welch		MH	
	OBs AV	DIF measure	DIF S.E	OBs AV	DIF measure	DIF S.E			t	*p*	Chi-sq	*p*
SP1	2.85	− 1.07	0.08	2.72	− 0.95	0.08	− 0.12	0.11	− 1.03	0.302	1.52	0.218
SP2	1.93	0.49	0.07	1.82	0.56	0.07	− 0.06	0.10	− 0.60	0.546	0.20	0.654
SP3	2.19	0.08	0.07	2.13	0.07	0.08	0.01	0.10	0.10	0.918	0.02	0.882
SP4	1.89	0.55	0.07	1.93	0.39	0.07	0.17	0.10	1.61	0.108	1.85	0.174
SP5	1.91	0.53	0.07	1.84	0.53	0.07	0.01	0.10	0.06	0.952	0.02	0.882
SP6	2.51	− 0.46	0.07	2.50	− 0.55	0.08	0.09	0.11	0.88	0.378	0.44	0.506
SP7	2.75	− 0.89	0.08	2.74	− 0.98	0.08	0.10	0.11	0.87	0.383	0.97	0.325
SP8	1.77	0.75	0.07	1.58	0.95	0.08	− 0.20	0.10	− 1.90	0.058	2.97	0.085
WR9	2.87	− 0.63	0.08	2.78	− 0.54	0.09	− 0.09	0.12	− 0.72	0.472	0.27	0.603
WR10	2.78	− 0.44	0.08	2.76	− 0.51	0.09	0.07	0.12	0.60	0.551	0.60	0.439
WR11	2.31	0.52	0.08	2.36	0.30	0.08	0.22	0.11	1.93	0.054	1.93	0.165
WR12	2.10	0.93	0.08	2.03	0.94	0.08	0.00	0.11	− 0.03	0.974	0.00	0.983
WR13	2.87	− 0.62	0.08	2.74	− 0.47	0.09	− 0.14	0.12	− 1.21	0.226	0.59	0.443
WR14	2.56	0.03	0.08	2.48	0.07	0.08	− 0.04	0.12	− 0.31	0.753	0.31	0.580
WR15	2.62	− 0.10	0.08	2.53	− 0.04	0.08	− 0.06	0.12	− 0.48	0.628	0.14	0.707
WR16	2.44	0.27	0.08	2.38	0.25	0.08	0.02	0.11	0.14	0.886	0.02	0.901
RE17	2.04	− 0.45	0.08	2.12	− 0.46	0.08	0.01	0.11	0.08	0.934	0.57	0.450
RE18	1.83	− 0.05	0.08	1.82	0.12	0.08	− 0.17	0.11	− 1.45	0.146	3.81	0.051
RE19	1.66	0.28	0.08	1.69	0.39	0.08	− 0.11	0.12	− 0.94	0.347	0.79	0.373
RE20	1.66	0.29	0.08	1.77	0.23	0.08	0.06	0.12	0.50	0.620	0.00	0.981
RE21	2.10	− 0.59	0.08	2.19	− 0.61	0.08	0.03	0.11	0.25	0.802	0.17	0.678
RE22	1.59	0.42	0.08	1.69	0.38	0.08	0.05	0.12	0.39	0.693	0.40	0.528
RE23	2.13	− 0.64	0.08	2.26	− 0.75	0.08	0.10	0.11	0.90	0.370	0.45	0.505
RE24	1.44	0.74	0.08	1.53	0.70	0.08	0.03	0.12	0.29	0.769	0.97	0.324
LI25	1.52	0.08	0.07	1.54	0.01	0.07	0.07	0.10	0.68	0.498	0.23	0.630
LI26	2.11	− 0.86	0.07	2.20	− 1.03	0.07	0.17	0.10	1.70	0.089	2.40	0.122
LI27	1.26	0.49	0.07	1.24	0.51	0.08	− 0.02	0.11	− 0.23	0.816	0.44	0.506
LI28	1.32	0.4	0.07	1.23	0.52	0.08	− 0.12	0.11	− 1.09	0.276	1.03	0.310
LI29	1.35	0.34	0.07	1.34	0.34	0.08	0.00	0.11	0.03	0.972	0.07	0.791
LI30	1.44	0.20	0.07	1.45	0.16	0.08	0.05	0.10	0.45	0.656	0.65	0.422
LI31	1.53	0.05	0.07	1.48	0.11	0.08	− 0.06	0.10	− 0.60	0.550	0.81	0.368
LI32	2.01	− 0.69	0.07	1.92	− 0.60	0.07	− 0.1	0.10	− 0.98	0.330	0.61	0.436

*OBS AV* Observations Average

**Fig. 4:**
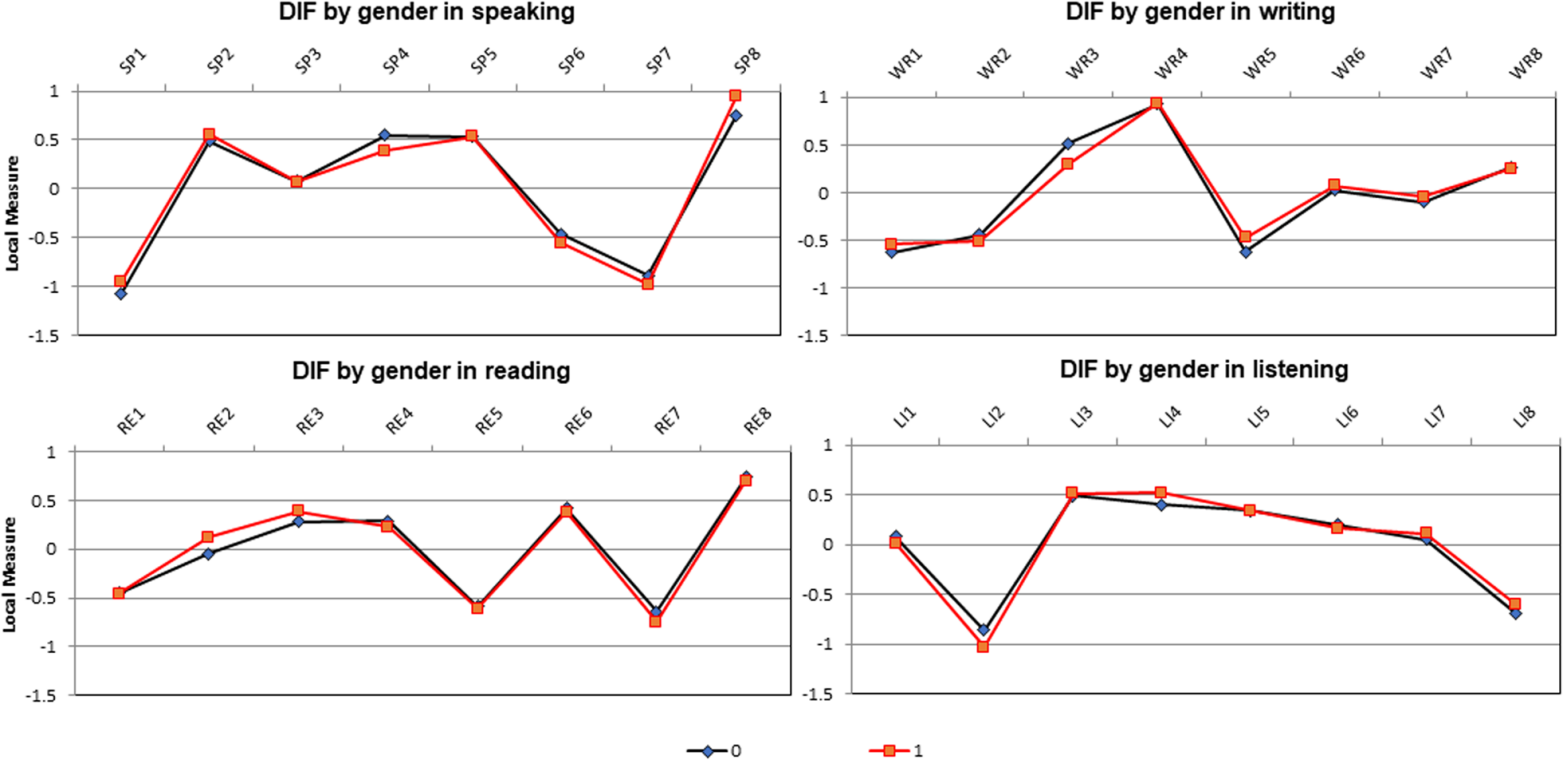
DIF local measures across sex in the four subscales

Figure 4 reflects the value of the item DIF in relation to the overall "baseline" item difficulty for the person-classification (by sex). This figure supports the results of Rasch–Welch’s t and M-H methods as it shows the plots of items which fall between 1 and -1 on ability measure for the four subscales (Table 5). Table 6 shows the non-significant differences between both sexes in the sub-scales and total score of the ELAS.

**Table 6:** Sex differences in ELAS subscales and total score, *N* = *604*

	Sex	M	SD	t	P
*Speaking anxiety*	Males	25.41	6.39	− .80	.425
	Females	25.81	6.09		
*Writing anxiety*	Males	28.05	4.99	− 1.21	.228
	Females	28.55	5.16		
*Reading anxiety*	Males	22.97	5.43	1.17	.243
	Females	22.45	5.64		
*Listening anxiety*	Males	20.40	6.15	− .28	.783
	Females	20.54	5.89		
*ELAS total*	Males	96.83	17.58	− .36	.717
	Females	97.34	17.15		

DF = 602

## Discussion

### Rasch analysis results

The Rasch model is characterized by robustness and objective assessment of latent traits [39], which is why this model was chosen to analyse the ELAS. The Results of the present study indicate that ELA is a multi-dimensional construct, and recent data support these findings [4, 5, 44]. The main aim of this paper was to examine the psychometric properties of the ELAS using the Rasch rating scale model and then detecting the DIF across sex. The overall results suggest that the ELAS shows good fit indices, so it is a proper instrument for the measurement of ELA in the Egyptian context.

In the present study, PCA indicated that there are four factors that explain students’ performance on the scale, which indicates its multi-dimensionality. Local independence was proved through the MNSQ fit, all of which fall within the acceptable range (see Table 4). However, IRT model parameters are robust enough and not greatly affected by violations in unidimensionality and local independence. As a result, we decided to retain all items and persons. After scrutinizing the MNSQ infit statistics, we found that all values are within acceptable levels (0.65:1.58). The overall findings of the Rasch analysis provide strong evidence that the ELAS is a promising measure of ELA in the Egyptian context.

According to [39], item misfit indicates that those items measure something other than or in addition to the targeted latent trait or construct of interest. Fortunately, all items of the ELAS show good fit. Based on the Rasch analysis, the ELAS can be regarded as a valid and reliable tool for the objective measurement of anxiety experiences in English language classes, because it showed high adherence to Rasch model assumptions. The provision of such a scale may help in collecting sound and accurate data, which in turn can help researchers in getting deeper insight into the ELA construct.

Because no DIF was detected for any item, the ELAS items do not appear to differently favour males over females or vice versa. The MH results indicate that the ELAS functions equally for both sex groups. Being calibrated by the Rasch model and bias free, the scale can be used in establishing standardized, meaningful and valid comparisons between sexes. This finding is contradictory to that of Saghafi et al. [7] who detected DIF in three items in the FLCAS and concluded that 23 out 26 items can be scored and interpreted in the same way for both sexes without the need to use different criteria. Similarly, Ra and Rhee [47] found that two items in the FLCAS exhibiting DIF for sex and claimed that the findings of previous studies related to sex differences in FLA might be attributed to the existence of DIF and not to real sex differences. In our study, ELAS registered no evidence of DIF across sex groups, so the scale can be reliably used with both sexes.

### Sex results

The non-significant differences between males and females indicate similar levels of ELA among the participants. This finding may be explained in light of the unified Egyptian system of education. It is possible that male and female Egyptian students have been educated so that they acquire and practise the same English language skills in similar ways, such that males and females experience approximately similar feelings of FLA. This resemblance may be attributed to the fact that both sexes consider English a major matter for their future career, not just a certificate: that is why they feel anxious during English classes. Mixed-sex classrooms and lack of self-confidence may represent an ELA-provoking situation and provide a possible reason for the similar experiences of ELA in both sexes. Insignificant sex differences accord with the findings of previous relevant studies [12, 32, 48, 58]. Another possible interpretation for the non-significant differences between males and females may be due in part to the nature of anxiety itself as a human trait inherent in personality and ELA is a situation-specific type of anxiety. The present results were inconsistent with the findings reported by [15, 55, 56] who found that females tended to be more anxious than males. On the other hand, Zhang [30] and Campbell and Shaw [80] found that males were more anxious than females in foreign language classrooms.

## Limitations and future research

Although the present measure was developed in light of IRT, which is more accurate and objective than the CTT, these results should not be taken for granted, because this research presents some limitations. First, the participants were selected from only one College of Education at only one university, and the relatively small sample size may result in a lack of generalizability for the results. Second, measure administered used a self-report scale. Future research should adopt other qualitative methods of data collection besides the self-report questionnaires. Future studies should also be conducted to detect the reasons and factors leading to ELA, and further intervention studies are needed to reduce the levels of ELA among college students. Assessment of ELA in pre-university institutions (primary, middle and high schools) needs to be investigated. Curriculum-based measurement is another promising trend in the diagnosis and alleviation of ELA that needs further research. We relied on the DIF detection methods provided by WINSTEPS software, meanwhile since the MH-statistic might be sensitive to uniform DIF, further research might double the sample size and reinvestigate the DIF across sex.

Qualitative research is needed to provide deeper insight into the personal and contextual factors causing ELA. Additionally, multiple case studies could be used as the basis for comparison and contrasting ELA within a cross-cultural perspective. The results of this research might open new horizons that would help future research inspire additional innovations within the field of ELA measurement. In summary, the given scale represents a promising measure of ELA among university students in general and in the Egyptian context in particular. The ELAS displayed strong adherence to the assumptions of the Rasch model. Notwithstanding its robust psychometric properties, the ELAS needs to be administered to diverse age, ethnic and culture groups for further scale assessment.

The findings of the present study can inform teachers, stakeholders and teachers about the current state of ELA among Egyptian undergraduates. Further studies in ELA among elementary school students are still required. Future research should investigate the DIF within a cross-cultural perspective. Scrutinizing the present findings, we can recommend that teachers and professors interested in assessing students’ ELA prior to course delivery can use the ELAS, because it appears to be a valid and reliable measure.

## Conclusions

This paper presents a promising ELA instrument consisting of 32 items measuring 4 subscales. It is intended to assess anxiety of learning English as a foreign language in Egyptian context. The scale turned out to be adequately valid and reliable in addition to its potential for precise use in comparison between males and females because it is invariant across sex. Given the satisfactory psychometric properties, the scale can be used in research and practice purposes.

## Data Availability

The datasets analyzed during the current study are available in the following link: https://drive.google.com/file/d/1dXfUUJCJC9dduIhBU8qtl83DziL2HoWx/view?usp=sharing.

## Abbreviations

FLA: Foreign language anxiety
ELAS: English language anxiety scale
EFL: English as a foreign language
ELA: English language anxiety
CTT: Classical testing theory
IRT: Item response theory
FLCAS: Foreign language anxiety scale
MH: Mantel–Haenszel
EFA: Exploratory factor analysis
CFA: Confirmatory factor analysis
PCA: Principal component analysis
SD: Standard deviation
GFI: Goodness of fit indices
RMR: Root mean square residuals
CFI: Comparative fit index
RMSEA: Root mean square error of approximation
TLI: Tucker Lewis index
SR: Standardized residuals
PCASR: Principal component analysis of standardized residuals
